# XPA-Mediated Regulation of Global Nucleotide Excision Repair by ATR Is p53-Dependent and Occurs Primarily in S-Phase

**DOI:** 10.1371/journal.pone.0028326

**Published:** 2011-12-12

**Authors:** Zhengke Li, Phillip R. Musich, Moises A. Serrano, Zhiping Dong, Yue Zou

**Affiliations:** Department of Biochemistry and Molecular Biology, East Tennessee State University, J. H. Quillen College of Medicine, Johnson City, Tennessee, United States of America; The University of Hong Kong, Hong Kong

## Abstract

Cell cycle checkpoints play an important role in regulation of DNA repair pathways. However, how the regulation occurs throughout the cell cycle remains largely unknown. Here we demonstrate that nucleotide excision repair (NER) is regulated by the ATR/p53 checkpoint via modulation of XPA nuclear import and that this regulation occurs in a cell cycle-dependent manner. We show that depletion of p53 abrogated the UV-induced nuclear translocation of XPA, while silencing of Chk1 or MAPKAP Kinase-2 (MK2) had no effect. Inhibition of p53 transcriptional activities and silencing of p53-Ser15 phosphorylation also reduced the damage-induced XPA nuclear import. Furthermore, in G1-phase cells the majority of XPA remained in the cytoplasm even after UV treatment. By contrast, while most of the XPA in S-phase cells was initially located in the cytoplasm before DNA damage, UV irradiation stimulated bulk import of XPA into the nucleus. Interestingly, the majority of XPA molecules always were located in the nucleus in G2-phase cells no matter whether the DNA was damaged or not. Consistently, the UV-induced Ser15 phosphorylation of p53 occurred mainly in S-phase cells, and removal of cyclobutane pyrimidine dimers (CPDs) was much more efficient in S-phase cells than in G1-phase cells. Our results suggest that upon DNA damage in S phase, NER could be regulated by the ATR/p53-dependent checkpoint via modulation of the XPA nuclear import process. In contrast, the nuclear import of XPA in G_1_ or G_2_ phase appears to be largely independent of DNA damage and p53.

## Introduction

The human genome is under constant threat of damage from exogenous genotoxic pollutants and carcinogens. Removal of DNA damage requires the dual action and coordination of cell cycle checkpoints and DNA repair machineries in each phase of the cell cycle [1]. The nucleotide excision repair (NER) pathway is the primary mechanism in cells for the removal of helix-distorting, replication-blocking DNA adducts induced by exogenous agents such as UV radiation and a variety of genotoxic chemicals [2]. In humans, defects of NER lead to the clinical disorder *Xeroderma pigmentosum* (XP) which is characterized by an increased sensitivity to UV radiation and a predisposition to the development of skin cancers [3], [4]. It remains elusive how NER is regulated by DNA damage checkpoints throughout the cell cycle.

The Xeroderma pigmentosum group A protein (XPA) is one of eight factors that were found to be deficient in XP disorders [5], [6], and the XPA-deficient cells exhibit the highest UV sensitivity among the XP cells [7]. XPA is an indispensable factor for both the transcription-coupled NER (TC-NER) and global genome NER (GG-NER) [8], [9]. NER can be regulated by transcriptional and post-transcriptional control of the XPA protein [10], [11], [12]. Functionally, XPA is believed to play roles in verifying DNA damage, stabilizing repair intermediates, and recruiting other NER factors to the damage site [13], [14], [15].

The DNA damage checkpoints survey the structural integrity of genomic DNA and coordinate multiple cellular pathways to ensure timely and efficient removal of DNA damage. The ATM (ataxia telangiectasia mutated)- and ATR (ATM- and RAD3-related)-mediated checkpoint pathways are two major genome surveillance systems in human cells. Both ATM and ATR are protein kinases belonging to the phosphoinositide 3-kinase-like kinase (PIKK) family. These pathways are comprised of a series of DNA damage sensors, signal mediators and transducers, and downstream effectors [1], [2], [16]. Checkpoint kinase-1 (Chk1), p53, and MAPKAP Kinase-2 (MK2) are the three main downstream checkpoint proteins that can be directly or indirectly activated by ATR following UV irradiation [17], [18], [19].

ATR can be activated by genotoxic agents that cause replication stress associated with accumulated RPA (Replication Protein A)-coated ssDNA [20]. In our previous studies, we found ATR and its kinase activity to be required for modulating translocation of cytoplasmic XPA into the nucleus upon UV-DNA damage [21]. Consistently, ATR was reported to be required for maintaining NER activity primarily during S phase in human cells [22]. When XPA translocation is inhibited by disruption of the ATR-XPA interaction in the nucleus, DNA repair efficiency is significantly reduced [23]. Regulation of nuclear import is necessary for timely localization of the repair proteins that participate in DNA repair [24]. These findings lead us to propose that ATR regulation of the XPA nuclear import may directly coordinate the ATR checkpoint activity with NER. However, the question as to whether the ATR-regulated nuclear import of XPA upon DNA damage is cell-cycle specific remains to be addressed.

In the current work, we demonstrate that UV-induced XPA nuclear import is cell cycle dependent and happens primarily in the S-phase, which may contribute to the ATR-regulated NER process. We also identified p53 as the ATR-regulated downstream protein required for the UV-induced XPA nuclear import and the removal of UV-DNA damage.

## Methods

### Tissue culture, drugs and antibodies

The A549/LXSN (p53+) and A549/E6 (p53−) cells were gifts from Dr. Jeffrey L. Schwartz [25]. Cells were maintained in D-MEM supplemented with 10% FBS and 1% penicillin-streptomycin. All cell lines were grown at 37°C, 5% CO_2_. UV-C irradiation was performed using a 254 nm lamp at a flounce of 0.83 J/m^2^/sec. For time course analysis cells, were incubated at 37°C, 5% CO_2_ for the indicated amounts of time. For inhibition of p53 transcriptional activities, pifithrin-α (Sigma Chemical Co.) was added into the culture medium at 30 µM and the cells incubated for 20 hours. For Western blotting, primary rabbit polyclonal antibody against XPA, mouse monoclonal antibody against PARP, rabbit polyclonal antibody against p53, mouse monoclonal antibody against Chk1, and goat anti-MK2 antibody were purchased from Santa Cruz Biotechnology Co. A FITC-conjugated primary mouse anti-actin antibody was obtained from Sigma Chemical Co. The anti-actin and anti-PARP antibodies were used in Western blots to confirm successful subcellular fractionations and protein loadings.

### RNAi and transfection

p53 and XPA siRNA duplexes were purchased from Santa Cruz. MK2 siRNA and Chk1 siRNA duplexes were synthesized by Genepharam using the following sequences: MK2 siRNA, sense strand 5′-UGACCAUCACCGAGUUUAUdTdT-3′ and antisense strand 5′-AUAAACUCGGUGAUGGUCAdTdT-3′; Chk1 siRNA, sense strand 5′-ACAGUAUUUCGGUAUAAUATT-3′ and antisense strand 5′-UAUUAUACCGAAAUACUGUTG-3′. The p53 3′-UTR siRNA duplexes were purchased from QIAGEN Corporation. Plasmids of human wild-type p53 and the Ser15Ala p53 mutant were gifts from Dr. Karen Vousden at the Beatson Institute for Cancer Research, United Kingdom. The co-transfection of p53 3′-UTR siRNA and p53 plasmids was done with Lipofectamine™ 2000 (Invitrogen) by following the company's instructions. The siRNA transfection reagent was purchased from Polyplus Transfection and were used following their instructions. Briefly, cells were grown to 30–40% confluence and washed with FBS- and antibiotic-free medium. siRNA duplexes were added to a small volume of FBS- and antibiotic-free medium and incubated with transfection reagent for 10 min. This siRNA/reagent mixture then was added to cells in FBS- and antibiotic-free medium at a final siRNA concentration of 5–10 nM. After 5–7 hours incubation, concentrated FBS and antibiotic medium were added into the transfection medium for further incubation. The cells were UV-irradiated at either 48 or 72 hrs of post-transfection. For time course experiments, siRNA-containing medium was removed for UV irradiation and added back for further cell growth.

### Immunoblotting

Cells were harvested by scraping or trypsin digestion, and re-suspended in lysis buffer (50 mM Tris-HCl, pH 7.8, 150 mM NaCl, 1 mM EDTA, 1% Triton X-100, 1× protease inhibitor cocktail [Roche]). Then 2× SDS loading buffer was added to the lysates and the mixtures were heated at 90°C for 10 min to denature proteins. After running the samples in SDS-PAGE, proteins were transferred from the gel onto a PVDF membrane. The membranes then were blocked with 5% nonfat milk in TBST and probed with specific primary and secondary antibodies. Chemiluminescence signal was captured with a Fuji Film LAS-4000 camera, and Western blot images were processed with Multi-Gauge 3.0 software.

### Cell synchronization, flow cytometry and BrdU incorporation assay

Cells were synchronized by mitotic “shake off” as described previously [26]. A549 cells were synchronized by seeding cells into four 300 cm^2^ flasks and grown to ∼70% confluence. Mitotic cells were collected by physically shaking the flasks to dislodge the loosely attached cells with monitoring by phase contrast microscopy at 20× magnification. For synchronizing HeLa cells, the cells were cultured in four 300 cm^2^ flasks and treated with nocodazole at 100 ng/ml for 8 hours (to enrich the mitotic cells) before shaking off. Collected mitotic cells then were seeded into 12-well or 6-well plates at 30–40% confluence and left to grow in standard culture condition. The synchronization was confirmed using BrdU labeling at each 4-hr time point after “shake off” and by flow cytometric cell cycle analysis after propidium iodide staining of the nuclear DNA.

For propidium iodide staining, cells were fixed in 70% cold ethanol for 1- to 16-hrs at 4°C. Fixed cells were centrifuged at 10,000×g for 10 sec to pellet the cells, and propidium iodide solution (PBS with 20 ug/mL propidium iodide and 100 ug/mL RNase (Invitrogen)) was added to re-suspend the cell pellet. The resuspended cells were incubated for another 30 min at 37°C. Stained cells then were analyzed using an Accuri C6 flow cytometer or a BD Biosciences flow cytometer to assess the DNA content. The cell synchronization results were processed by FCS software.

BrdU incorporation was performed following the company's instructions (Cellomics) with a few modifications. Briefly, cells were grown on coverslips and labeled with BrdU by adding the nucleoside to a final concentration of 160 µM; the cells were grown for an additional 15 min to label those in S phase. Cells then were fixed with 4% paraformaldehyde solution before treatment with permeabilization buffer. After blocking with 15% BSA for 1 hr at room temperature, primary mouse anti-BrdU antibody and fluorescence-conjugated secondary antibodies (Invitrogen) were used to detect BrdU incorporation. Stained cells were visualized using 100× magnification with fluorescence microscopy.

### Subcellular fractionation

Subcellular fractionation was performed using the Proteo JET™ cytoplasmic and nuclear protein extraction kit (Fermentas) by following the procedures suggested by the manufacturer. Briefly, 10 volumes of cell lysis buffer (with 1× protease inhibitors) were added to 1 volume of packed cells. After a short vortexing and incubation on ice for 10 min, cytoplasm was separated from nuclei by centrifugation at 500×g for 7 min at 4°C. Isolated nuclei were washed with 500 µL of the nuclei washing buffer and collected by centrifugation. The collected nuclear pellets were re-suspended in ice-cold nuclear storage buffer, and 1/10 volume of the nuclear lysis reagents was added to lyse the nuclei with rotation for 15 min at 4°C. Nuclear lysate was collected by centrifugation at 20,000×g for 15 min at 4°C. In all of the fractionation experiments, Western blotting of β-actin and PARP were assessed to check cytoplasmic and nuclear protein loading, respectively.

### Immunofluorescence microscopy

For immunofluorescence microscopy of proteins, cells were grown on coverslips before the initiation of experimental treatments. After UV-C irradiation and specified recovery times, the cells were fixed with 100% cold methanol and blocked with 15% BSA for 1 hr at room temperature. Proteins were detected with primary antibodies and fluorescence-conjugated secondary antibodies (Invitrogen). Cells on coverslips were coated with prolong gold antifade reagent containing DAPI (Invitrogen) before microscopic examination using 100× magnification.

### Slot-blot DNA Repair Assay

Cells were seeded at 1×10^6^ cells per 10 cm tissue culture dish and allowed to grow for the indicated periods prior to UV-C irradiation. After irradiation, cells were allowed to recover for the indicated periods, followed by genomic DNA purification using the QIAGEN DNA Mini Kit. The purified DNA was quantified by measuring the light absorbance at 260 nm and diluted to 0.2 µg/mL in a final volume of 200 uL TE buffer. The DNA was denatured by incubating at 90°C for 10 minutes, followed by rapid chilling on ice water before adding an equal volume of 2 M ammonium acetate. Samples were immobilized on a nylon membrane and probed using monoclonal mouse anti-CPD or anti-(6-4)PP antibodies (Kamiya Biomedical Co.).

### Statistical analysis

The statistical analysis of samples was performed with a two-tailed student's t-Test, and a p-value of less than 0.05 was considered as significant.

## Results

### UV-induced XPA nuclear import depends on p53

**Figure 1:**
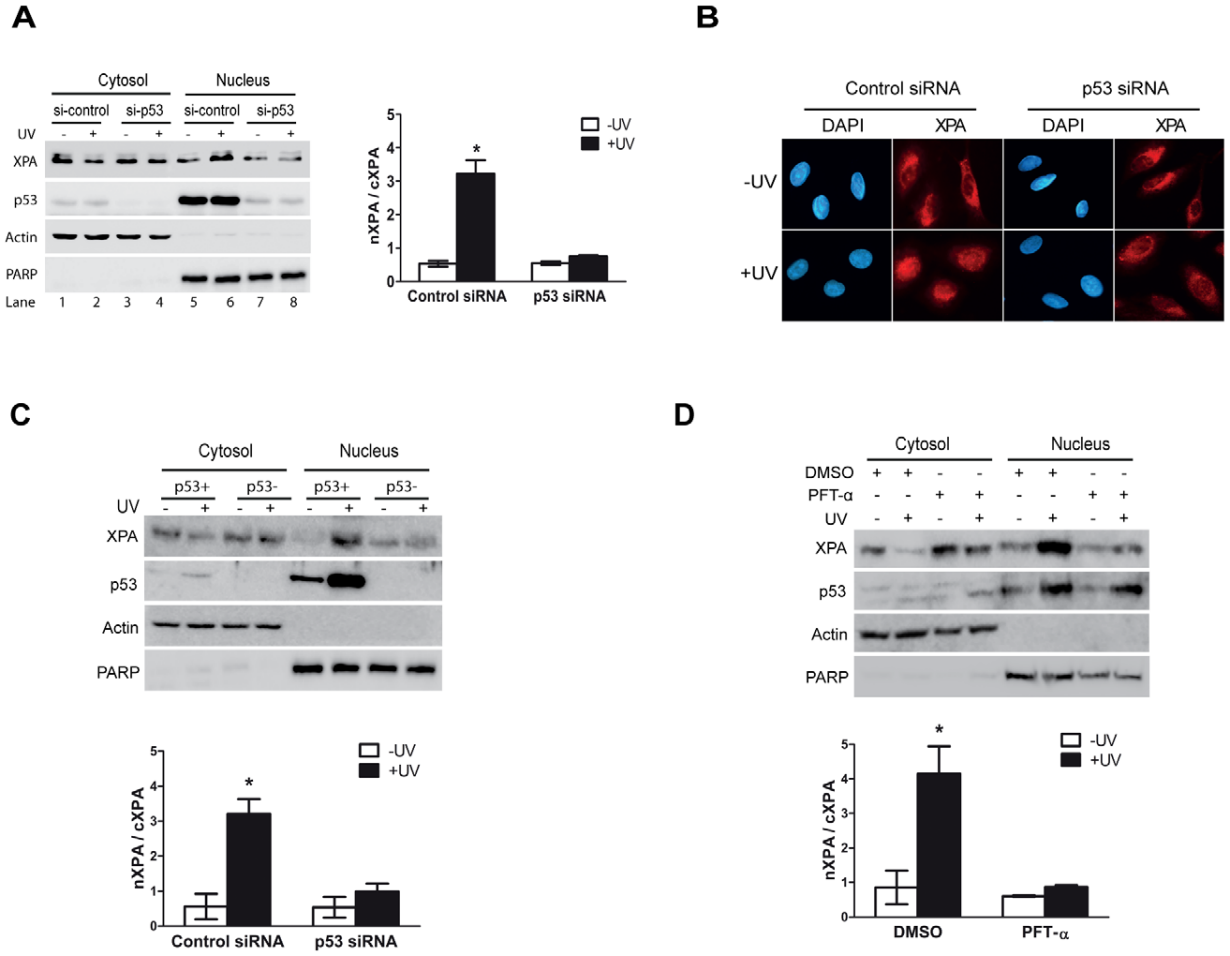
p53 is required for the XPA nuclear import upon UV irradiation. **A,** p53 was transiently knocked down with siRNA duplexes in HeLa cells. After treatment with or without 20 J/m^2^ UV followed by a 2-hr recovery, subcellular fractionation and Western blotting were performed to assess the re-distribution of XPA. β-actin and PARP were probed as cytoplasmic and nuclear protein controls, respectively. The quantitative data were obtained from at least three independent experiments. nXPA/cXPA represents the ratio of nuclear XPA to cytoplasmic XPA. **B,** Immunofluorescence microscopic analysis of cells transfected with control or p53 siRNA and with or without UV irradiation. **C,** A549/LXSN(p53+) and A549/E6(p53−) cells were mock- or UV-irradiated. Cytosol and nuclear fractions were collected and analyzed by Western blotting. **D,** A459 cells were pre-treated with pifithrin-α (30 uM), an inhibitor of p53 transcriptional activity, for 20 hrs. After UV irradiation and a 2-hr recovery, the cells were analyzed for subcellular localization of XPA. The * in the plots indicates a statistically significant (p<0.05) difference between the groups being compared.

We previously demonstrated that UV-induced XPA nuclear translocation is dependent on ATR [21]. Since p53 is a major downstream substrate of ATR, it is of interest to determine if p53 is required for XPA nuclear import. Thus, cells were transfected with p53 siRNAs. As shown in Figure 1A, the p53 silencing inhibited the UV-induced nuclear import of XPA. In the control siRNA-transfected cells, most of XPA molecules were imported into the nucleus after DNA damage (compare the nuclear XPA-to-cytoplasmic XPA (nXPA/cXPA) ratio in lanes 6 and 2 with the ratio in lanes 5 and 1; also see the adjacent plot). By contrast, the nXPA/cXPA ratio was significantly reduced in the cells with p53 depletion (Figure 1A). β-actin and PARP are cytoplasmic and nuclear proteins, respectively, and were probed as controls to indicate the quality of the cytoplasmic/nuclear protein fractionation. An immunofluorescence microscopy assay also was performed and the same effect of p53 on the XPA nuclear import in the cells was observed (Figure 1B). In the absence of DNA damage, most XPA molecules were located in the cytosol of the cells transfected with control siRNA, but were translocated into the nucleus following UV irradiation. However, only a small portion of cytosolic XPA was translocated into the nucleus in the p53-silenced cells even after UV irradiation. To further confirm these results, A549/E6 (p53−) and A549/LXSN (p53+) cells were employed. In A549/E6 (p53−) cells, p53 is abrogated due to overexpression of human papillomavirus type 16 E6 protein [25], while in A549/LXSN (p53+) cells wild-type p53 is expressed. As shown in Figure 1C, the UV-induced XPA nuclear translocation was disrupted in the p53-deficient cells as compared to the p53-proficient cells.

We next examined the effect of transcriptional function of p53 on XPA nuclear import. A549 cells were pre-incubated with pifithrin-α, a p53 transcriptional activation inhibitor, before the UV treatment. As shown in Figure 1D, the presence of pifithrin-α significantly reduced the UV-induced XPA nuclear import (the nXPA/cXPA ratio) as compared to the DMSO-treated cells. The data suggest that p53 may regulate the UV-induced nuclear import of XPA through its transcriptional activity.

### Neither Chk1 nor MK2 is required for UV-induced XPA nuclear import

**Figure 2:**
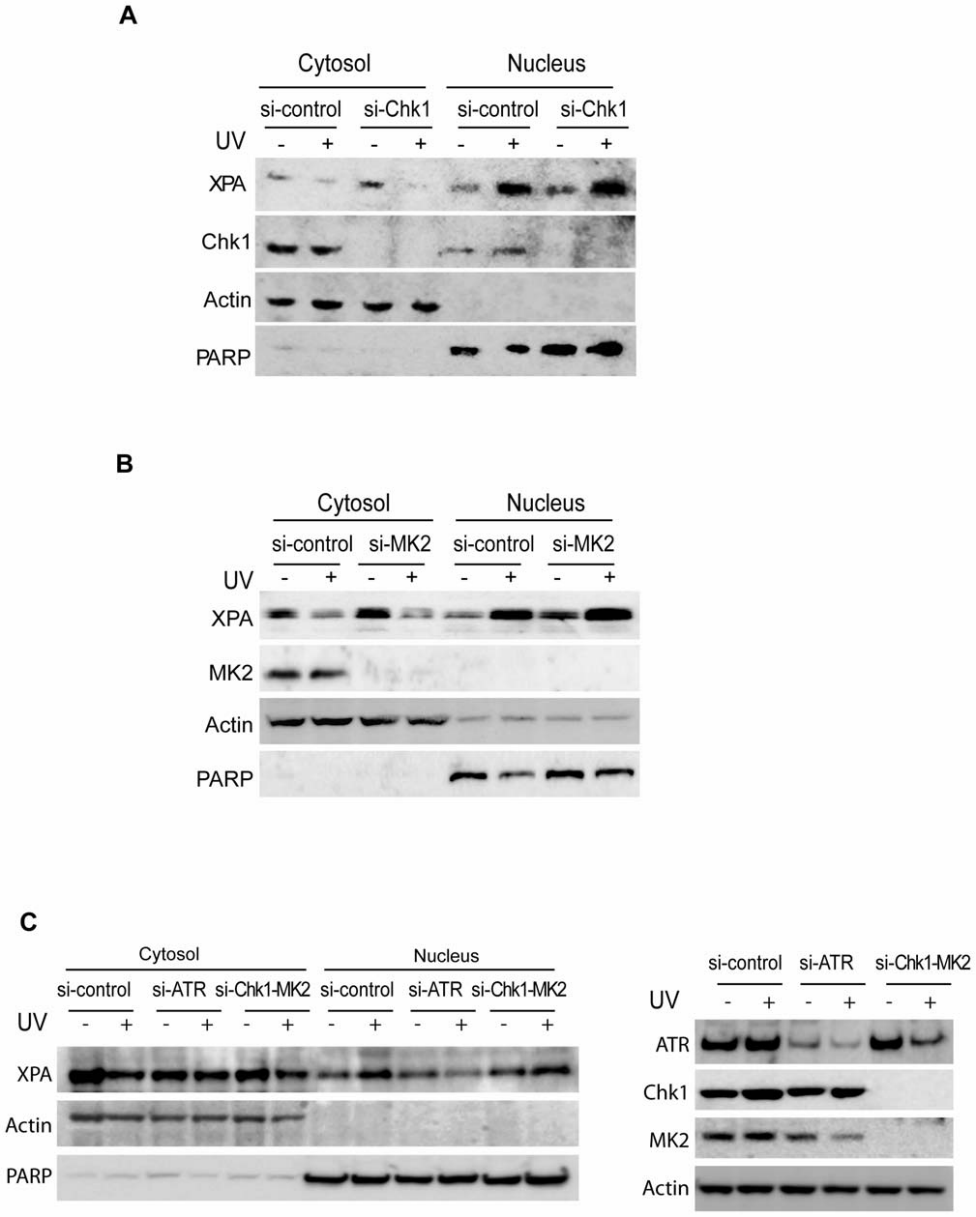
Cell cycle checkpoint proteins Chk1 and MK2 are not required in the UV-induced nuclear import of XPA. **A.** siRNA duplexes targeting Chk1 were transiently transfected into A549 cells, followed by mock or 20 J/m^2^ UV irradiation and a 2-hr recovery. The localization of XPA was assessed using subcellular fractionation followed by Western blot analysis. PARP and β-actin proteins were probed as nuclear and cytoplasmic protein controls, respectively. **B.** A549 cells were treated with MK2 or control siRNA, followed by UV irradiation. After a 2-hr recovery period, irradiated cells were fractioned and analyzed by Western blotting. **C.** Chk1 and MK2 were simultaneously knocked down by Chk1 and MK2 siRNAs, or ATR was knocked down by ATR siRNA. Then, the UV-induced XPA nuclear import in these cells was assessed by fractionation and Western blotting.

Chk1 is a downstream kinase substrate of ATR and may play an important role in transducing damage signals in the ATR pathway by phosphorylating p53. MAP kinase-activated protein kinase 2 (MAPKAPK2 or MK2) is another downstream kinase of ATR. The p38-MK2 pathway recently was identified as an alternative checkpoint in p53-deficient cancer cells [18]. It is of interest to investigate whether Chk1 and/or MK2 are involved in the UV-induced nuclear translocation of XPA. To this end, A549 cells were transfected with Chk1 or MK2 siRNA followed by subcellular fractionation. The results in Figures 2A and 2B indicate that neither Chk1 nor MK2 is required for the UV-induced XPA nuclear import as no difference of the import was observed between the control siRNA- and the Chk1 or MK2 siRNA-transfected cells. Similar results also were obtained for cells depleted of both Chk1 and MK2 (Figure 2C). By contrast, siRNA knockdown of ATR in the A549 cells did significantly reduce the UV-induced nuclear import of XPA (Figure 2C), confirming the involvement of ATR/p53 checkpoint pathway in the regulation of XPA nuclear import.

### UV-damage induced XPA nuclear import occurs primarily in S-phase

**Figure 3:**
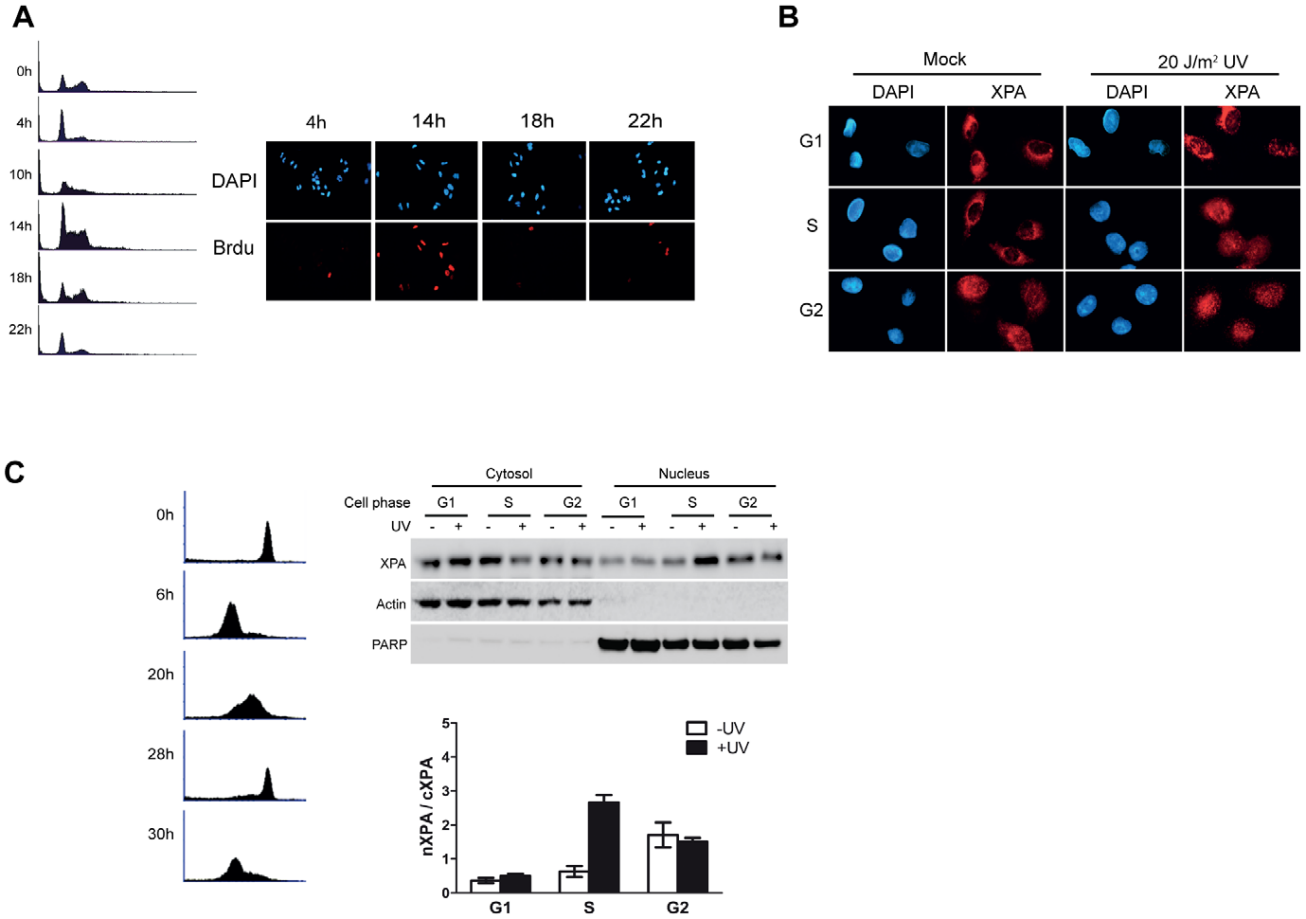
DNA damage-induced XPA nuclear accumulation occurs primarily in S-phase. **A.** Mitotically-synchronized A549 cells grown for the indicated time periods were stained with propidium iodide for analysis of the cell cycle distribution (Panel A, left) or labeled with BrdU to identify synchronized S-phase cells (panel A, right). **B.** Immunofluorescence microscopic analysis of the subcellular localization of XPA in the synchronized cells. Synchronized A549 cells were mock- or UV-treated (20 J/m^2^) and left to recover for 2 hrs. Cells were fixed and stained with primary and fluorescence–conjugated secondary antibodies to determine the localization of XPA. At least 100 cells were examined, and the representative data is shown. **C.** Left: Mitotically-synchronized HeLa cells were stained with propidium iodide followed by flow cytometric analysis. Right: Subcellular fractionation followed by Western blotting was performed to analyze the subcellular localization of XPA in each phase of the cell cycle after UV irradiation of synchronized HeLa cells. PARP and β-actin were probed as nuclear and cytoplasmic protein controls, respectively. At least three independent experiments were performed and representative data is presented.

Next we addressed the question of whether the ATR-dependent XPA nuclear import following DNA damage is cell-cycle specific as is the ATR checkpoint [27]. To avoid any physiological perturbation associated with drug treatments, A549 cells were synchronized by mitotic “shake off” as described previously [26]. The collected mitotic cells were seeded into cell culture dishes and maintained at standard culture condition to generate a synchronized cell population for subsequent analysis. The synchronization efficiency was assessed by propidium iodide (PI) staining followed by flow cytometric analysis and by assessing the BrdU-labeled cells under fluorescence microscopy (Figure 3A). Based on the results, the 4-hour post-“shake off” time point was selected as the G1 cell population (most cells with a 2C DNA content and the lowest level of BrdU labeling); the 14-hour time point was selected as the S-phase cell population (DNA content between 2C and 4C and highest BrdU labeling); the 18-hour post “shake off” time point was selected as the G2 cell population (4C DNA content and low BrdU labeling). The location of XPA molecules in the synchronized cell populations was assessed using immunofluorescence [23]. Figure 3B shows that in the G1 cells most of the XPA molecules were located in the cytosol, and there was only a slight accumulation of XPA in the nucleus after UV irradiation. By contrast, although most of the XPA molecules were located in the cytosol in S-phase before UV irradiation, they were imported into the nucleus after UV treatment. Interestingly, in the G2 cell population most cells showed significant levels of XPA in the nucleus either with or without UV irradiation.

To determine whether this cell phase-specific translocation of XPA is a general response, synchronized HeLa cells (Figure 3C, left) were treated with or without UV and then subjected to subcellular fractionation for analysis of subcellular localization of XPA in each phase of cell cycle. Consistent with the observation in the A549 cells, we found that in HeLa cells, the UV-induced XPA nuclear translocation also occurs primarily in the S-phase (20 hrs post “shake off”) (Figure 3C, right). Similarly, in the G1 phase cells (6 hrs post “shake off”), most XPA remained in the cytoplasm even after UV treatment, while in the G2 phase cells (28 hrs post “shake off”), a large amount of XPA was found in the nucleus even without UV damage.

### Repair of CPDs is significantly slower in G1 than in S phase

**Figure 4:**
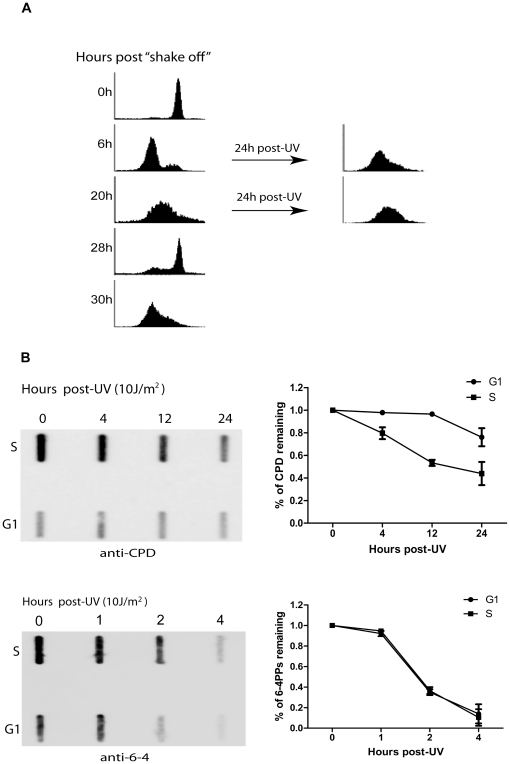
Removal of UV-induced DNA damage in G1- and S-phase cells. **A.** Mitotically-synchronized HeLa cells were fixed and stained with propidium iodide at the indicated time points following the mitotic “shake off”. The cell cycle distribution then was analyzed by flow cytometry. Cells at G1 (at the 6 hours post-“shake off”) or S (20 hours post-“shake off”) phase, were UV irradiated at 10 J/m^2^, followed by a recovery of 24 hours. **B.** Cells at G1 or S phase were UV irradiated at 10 J/m^2^, followed by the indicated periods of repair. Cellular DNA were isolated and the removal of CPDs and 6-4PPs was measured by slot-blot assay. The amounts of CPDs or 6-4PPs were normalized to the values at zero hour and quantified based on three independent measurements.

As described above, the UV-induced XPA import occurs primarily in S-phase cells, particularly in comparison with that in G1 phase (Most of the XPA is located in the nucleus in G2 phase even in the absence of DNA damage). Thus, it was expected that repair of the UV-induced DNA damage could be more efficient in S phase cells than in G1 phase cells. To confirm this, HeLa cells were mitotically synchronized and the cells in either G1 or S phase were UV-irradiated at a dose of 10 J/m^2^, followed by the indicated periods of recovery (Figure 4A). As shown in Figure 4B, more CPDs were generated in S phase than G1 phase cells following UV irradiation, likely due to the more open chromatin structures in S phase than in G1 phase (thus less protection of DNA from UV damage). As expected, the repair rate of CPDs was much higher in S phase than in G1 phase cells. In contrast, no difference in repair efficiency for 6-4PPs was observed between G1 and S phase cells (Figure 4B). It is well known that 6-4PPs can be removed in cells within a few hours while CPDs are the much persistent DNA damage that is responsible for the UV-induced cell death in NER proficient cells [28]. It also is known that 6-4PPs are the minor lesions induced by UV in cells as compared with CPDs [29], [30]. Since the repair of 6-4PPs is generally so efficient, it is possible that the relatively low level of XPA in the nuclei of G1 phase cells is adequate for efficient removal of the relatively small quantity of 6-4PPs.

### Phosphorylation of p53 on serine15 is required for the UV-induced XPA nuclear import

**Figure 5:**
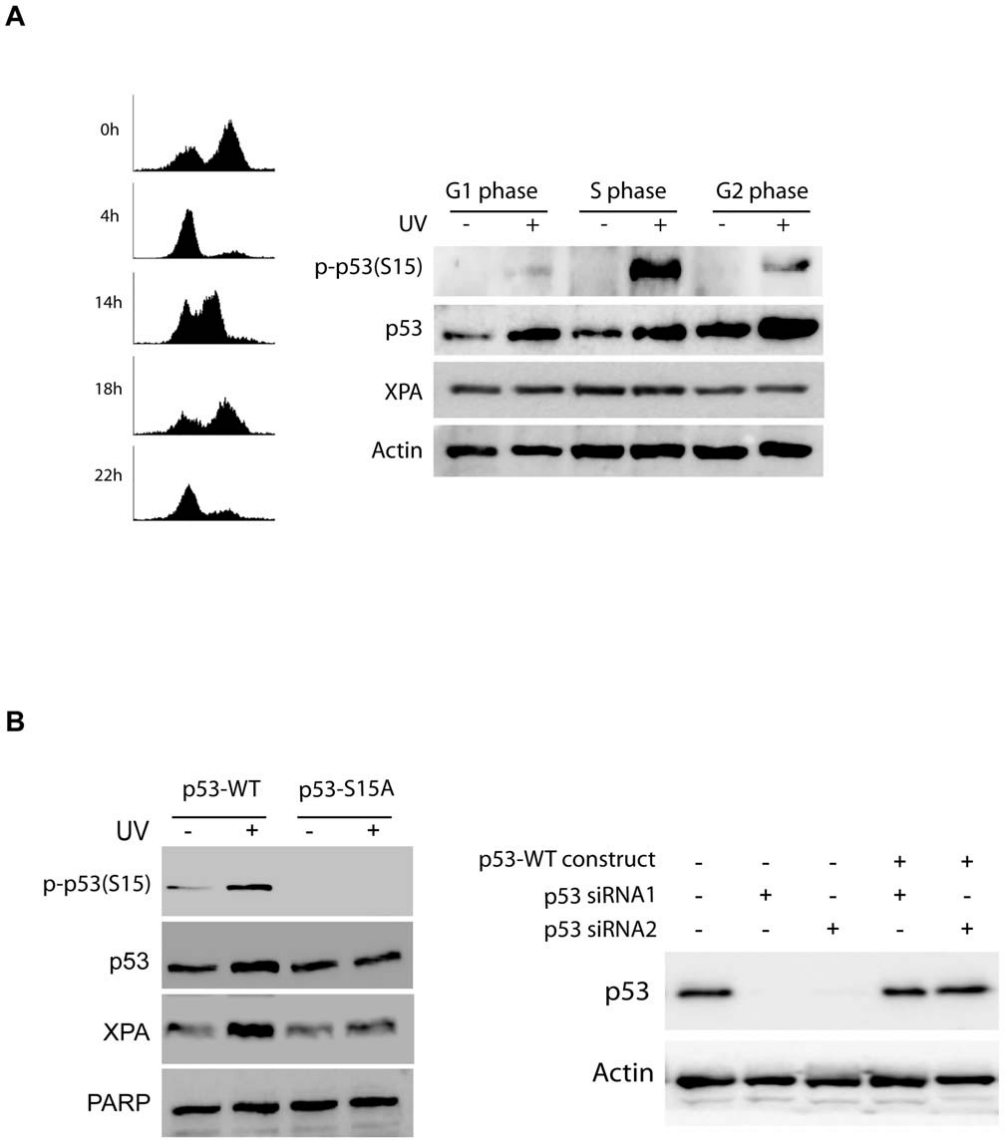
Phosphorylation of p53 is required for the UV-induced XPA nuclear import. **A.** Mitotically synchronized A549 cells were mock-treated or irradiated with 20 J/m^2^ of UV-C, and allowed a 2-hr recovery before accessing the phosphorylation of p53 at Ser15 by Western blotting. **B.** Constructs for expressing human wild-type p53 or the S15A mutant of p53 were co-transfected with p53 3′-UTR siRNA into A549 cells. 72 hours after transfection, the A549 cells were mock- or UV (20 J/m^2^)-treated and allowed a 2-hr recovery. The UV-induced phosphorylation of p53 and the XPA in the nuclear fraction then were analyzed by Western blotting. The right panel shows the efficiency of siRNA knockdown of endogenous p53 and the level of recombinant p53 in the cells co-transfected with p53 3′-UTR siRNA and p53-WT constructs.

Although our results showed that p53 is required for the XPA nuclear import (Figure 1), it is unclear whether the requirement involves the checkpoint activity of p53. Therefore, we examined the effect of the phosphorylation of p53 at Ser15 on XPA nuclear import. The p53 phosphorylation at Ser15 plays an important role in ATR-dependent checkpoint signaling [2], [19]. Consistent with the observed cell phase-specific nuclear import of XPA induced by UV, the UV-induced phosphorylation of p53 on Ser15 was found to occur predominantly in the S-phase cell population (Figure 5A). We next assessed the requirement of p53 serine15 for the UV-induced XPA nuclear import. For this purpose, the endogenous p53 in A549 cells was depleted using 3′UTR siRNA while the cells transiently expressed recombinant siRNA-resistant wild-type p53 or S15A-mutant p53. The deficiency of the Ser15 phosphorylation in the p53-S15A construct-transfected cells was confirmed by Western blotting (Figure 5B). Also shown in Figure 5B, the UV-induced XPA nuclear import in the cells expressing p53-S15A was significantly lower than in the cells expressing wild-type p53.

## Discussion

A precise regulation of DNA repair is essential for cells to function normally in response to DNA damage. Given the key role of XPA in NER, results from our previous [21], [23] and current studies suggest that the ATR-dependent regulation of the damage-induced XPA nuclear import may represent a novel mechanism by which NER activity can be regulated by DNA damage checkpoints. Here we found that this regulation occurs primarily in S-phase, which well reflects the fact that DNA is most vulnerable to insult in S-phase in terms of maintaining genome integrity. This also is consistent with the recent report that ATR kinase is required for GG-NER exclusively during S phase [22].

We also examined whether any of the major downstream checkpoint substrates of ATR such as p53, Chk1 or MK2, is involved in the regulation of the UV-induced XPA nuclear import. Our results indicate that the XPA nuclear import is dependent on p53 in cells responding to UV damage, but neither Chk1 nor MK2 are required for this XPA nuclear translocation (Figure 2). The results suggest a regulatory role of p53 in NER, which is in agreement with previous studies [31], [32], [33], [34], [35], [36]. We found that not only is the p53 protein itself necessary (as shown in the siRNA knockdown experiments), but also the transcriptional function of p53 and the damage signaling via p53-ser15 phosphorylation are required for the UV-induced XPA nuclear import (Figures 1 and 5). In fact, the Ser15 phosphorylation of p53 has been shown to stimulate the transcriptional functions of p53 through its increased association with p300 co-activator [37], [38], [39] and stabilization via disruption of binding to MDM2 [40]. Thus, the effect of p53-ser15 phosphorylation appears to converge with that of the p53 transcriptional activation inhibitor pifithrin-α. The Ser15 of p53 can be phosphorylated either directly by ATR or by ATR-activated Chk1 in response to UV irradiation [2], [17], [19], [41]. Since Chk1 was not required for UV-induced XPA nuclear import (Figure 2), ATR kinase may directly phosphorylate p53 for transcriptional activation of the XPA nuclear translocation. Given that p53 activates transcription of multiple genes involved in numerous cellular processes [42], it is possible that p53 may enhance the transcription of the genes involved in XPA trafficking from cytoplasm to nucleus [43], [44]. More recently, studies have showed that the transcription factor E2F1 plays a role in facilitating the recruitment of XPA and other NER factors to the UV-induced DNA damage sites and this appears to be mediated by ATM- and ATR-dependent phosphorylation of E2F1 at Ser31 [45], [46]. Evidently, future investigation is needed to identify the p53-regulated transcriptional targets involved in the protein nuclear import process.

In contrast to the case in S phase, most of the XPA molecules remained in the cytoplasm of G1-phase cells after UV irradiation, while XPA normally accumulated in the nucleus in G2 phase cells even without DNA damage. In addition, the level of UV-induced Ser15-phosphorylation of p53 was much lower in G1- and G2-phase cells than in S-phase cells (Figure 5A). These results suggest that the UV-induced XPA nuclear import happens predominately in S phase (Table 1), while the XPA nuclear import in G1 and G2 phases is largely, if not fully, independent of UV irradiation and p53. Consistently, DNA repair of UV-induced CPDs was much more efficient in S phase than in G1 phase (Figure 4). Also interestingly, it was previously demonstrated that p53 deficiency had a negative impact on GG-NER but not on TC-NER [31], [33]. Given the indispensable role of XPA in both GG-NER and TC-NER, the observation of a p53 requirement for the UV-induced XPA nuclear import in S-phase cells implies that the TC-NER may predominately occur in other cell cycle phases in a p53-independent manner. Consistently, the rate of transcription is generally low during S phase except the transcription for histone production.

**Table 1:** Subcellular localization of XPA in the presence and absence of UV irradiation.

Interphase	−UV		+UV	
	Cyto	Nucl	Cyto	Nucl
**G1**	+++++^a^	+	++++	++
**S**	+++++	+	+	+++++
**G2**	+	+++++	+	+++++

a The number of “+” indicates the proportional distribution of XPA in the cytosol or the nucleus at individual parts of the cell cycle.

Compared to the UV-induced XPA nuclear translocation in S phase, the subcellular distributions of XPA in the absence and presence of UV are very different in G1 and G2 phases. In typical human cells, G1 phase lasts much longer than S phase and the G1-phase cells do not experience a replication pressure requiring a fast repair of DNA damage. Thus, it is possible that the relatively lower rate of NER could be sufficient for timely removal of DNA damage by the end of G1 phase. In addition, the G1/S DNA damage checkpoint also could prevent cells from entering S phase before the damage has been removed. In the case of G2 phase, since XPA nuclear accumulation occurs even in the absence of DNA damage, the accumulation is not a residual effect of an S phase accumulation. This may imply a quick removal of DNA damage in G2. It may also implicate a possible role of XPA in G2 maintenance or G2/M checkpoint regulation of the normal cell cycle. Indeed, depletion of XPA protein in cells changed the population distribution of cells in the cell cycles (data not shown). Although the potential role of XPA in normal cell cycle is of interest, it is out of scope of this study and deserves further investigations.
